# P02.46. Double blind randomised controlled study of the acute (immediate) cardiovascular effects of reflexology in healthy volunteers and cardiac patients

**DOI:** 10.1186/1472-6882-12-S1-P102

**Published:** 2012-06-12

**Authors:** J Jones, P Thomson, W Lauder, S Leslie

**Affiliations:** 1 University of Stirling, Inverness, United Kingdom; 2 NHS Highland, Inverness, United Kingdom

## Purpose

Reflexology is one of the top six complementary therapies used in the UK. Reflexologists claim that massage to specific points of the feet increases blood supply to referred or ‘mapped’ organs in the body. Empirical evidence to validate this claim is scarce. This three-phase study measured changes in cardiovascular parameters in subjects receiving reflexology treatment applied to specific areas of the foot which are thought to correspond to the heart (intervention) compared with reflexology applied to other areas on the foot which are not (control).

## Methods

16 reflexology-naive healthy volunteers, 12 reflexology-naive patients with chronic artery disease (CAD) and 12 reflexology-naive patients with heart failure (HF) received active and control reflexology treatments in three randomised, placebo-controlled, double-blind repeated measures studies. In each study the subjects were observed over six time periods under the two conditions and randomised to receive either intervention or control treatment. Outcome measures included ‘Beat-to-beat’ non-invasive continuous measurement of heart rate, diastolic blood pressure, stroke volume, stroke index, cardiac output, cardiac index, total peripheral resistance, baroreceptor reflex sensitivity, and heart rate variability.

## Results

The effects of reflexology treatment were modest. There were no significant differences noted in any of the measured parameters in either the CAD or HF intervention or control groups. Cardiac index decreased significantly in the healthy volunteer intervention group during left foot treatment (LFT) (baseline mean 2.6; (SD) 0.75; 95% CI +/- 0.38 vs. LFT mean 2.45; SD 0.68; CI 0.35) with an effect size (p= 0.035, omega squared effect (w2) = 0.002; w = 0.045).

## Conclusion

The findings suggest that reflexology massage applied to the upper part of the left foot in the area thought to relate to the ‘heart’ may have a modest specific effect on the cardiac index of healthy volunteers, but no specific effect on patients with various gradations of cardiovascular disease.

